# The Province of Psychology

**Published:** 1862-04

**Authors:** W. G. Davies


### Art. III.—THE PROVINCE OF PSYCHOLOGY.

By Rev. W. G. Davies.

Few things have so greatly impeded the formation of a practical science of mind, few have contributed so much to the confusion of mental philosophy, as the dissidence of opinion existing between, and the long-standing rivalry of, psychologists and mental physiologists. The former pursuing their researches according to the reflective method, the latter according to the method of physical science, both classes of investigators have too commonly maintained that they had nothing in common the one with the other; both have too readily assumed that their methods and results were incompatible and irreconcileable, and that there was no necessity of intimate connexion between them. Both, indeed, have magnified their half-systems into whole ones, and have held that their two-sided truths were nothing less than four-square.

The idealist, for he *par excellence* is your downright psychologist, wholly absorbed with the processes of thought, with the world of knowing, proposes to discover how we know, with a view of ascertaining what it is that we can be said, without the slightest approach to fiction, most truly to know. Anxious to fix our knowledge on a sure foundation which no scepticism can overthrow, desiring to eliminate from philosophy every element of faith, or, what is founded on extrinsic authority, he analyses consciousness with that laudable end in view. This basis he lays, as he thinks, perfectly secure, and it is only when, relying with entire confidence on this presumed security, he builds upon it a most grotesque and unnatural superstructure, that the impartial observer is tempted to conclude, that principles which lead to such strange consequences must have a grave flaw lurking somewhere in them. Careful not to proceed but where his feeble light enables him, the idealist, in common with all those who, wedded exclusively to a demonstrative system, are indisposed to admit anything as true which is not established either by observation or by proof, is confined to a very narrow field of research. Now, it is evident that before knowledge of any subject reaches a demonstrative, scientific, or final stage, it must previously pass through, at least, two stages—a spontaneous stage, and a transitional one. The demonstrative inquirer too frequently limits knowledge to what is within his own clear, but confined view. He forgets that before knowledge can come to him in an acceptable shape, it must pass through certain preliminary processes. He is but the finisher of other men's work, yet he would have the iron ready to work into rails, or into Armstrong guns, without acknowledging the necessity of those preparatory processes by which it is divided from the ore. Although we procure more correct knowledge by this stringent method, yet by a bigoted adherence to it, our mental horizon gets sadly circumscribed, a cold shade of negation chills spontaneous thought, and frowns down the exercise of a wise and pleasant faith. And, indeed, by adopting such a method, and by relying too implicitly on the presumed certainty of our first principles, we may be led into the elaboration of a system which has not one feature to recommend it to the acceptance of those who possess acute instinctive apprehensions of what is true, but are unable to prove what they believe to be so. The part of true wisdom is to accept, not merely what is demonstrated, but, as we are taught by the great Coryphæus of the Philosophy of Common Sense—in the spirit at least of his writings—to open wide the portals of the mind to those spontaneous and transitional thoughts and theories which invariably precede a final or scientific stage of knowledge. A grand and powerful inducement for cultivating this temper of mind is, that the superior truths are those which enter last upon their final growth, and are, consequently, at this present moment demanding from us the exercise of a large-hearted faith.

The mental physiologist, on the other hand, is equally as much of an enthusiast as the idealist, and ambitiously claims the whole man, body and soul, as his province. He adopts the *à posteriori* method. He is not so anxious about ascertaining absolute certainty, as about constructing a system that will satisfy the practical intelligence of those who are skilled in his department, the enlightened common sense of his peers. Psychology he rails at as an absurd method. 'What truth can a man discover by pondering over his own consciousness, and drawing metaphysical cobwebs out of his own mind? If all men possessed similar mental conformations, there might be some hope of arriving at trûth by this solitary converse with self. The precept, Know thyself, would then indeed point out the way to the true philosophy of the human mind. But there is as much variety among men in respect to mental constitution, as there is between them in respect to stature, hue, and feature. No, a sound mental science can only be established by the physiological method, by observation, by a strict adherence to facts as they are. *A priori* speculation, as a method of constructing mental science, has been tried, and has signally failed. Psychology proper exhibits a most deplorable state of anarchy. Were a man to propose selecting his mental philosophy from among the many conflicting systems which compete for his favour, he would, indeed, be sadly at his wit's end, as to which he should choose.

Somewhat in the above style the extreme cerebral physiologist estimates the labours of the reflective school of thought. There are, however, some men of an eclectic spirit, who endeavour to hold the balance between the rival physiological and psychological methods, and upon the success of their efforts the future advancement of mental philosophy and the possibility of forming a practical science of mind, will, we believe, chiefly depend. We propose to illustrate briefly this view of the question.

The psychologist strictly confines himself to the phenomena of consciousness. These he can know only by reflection, or by introspection. Of that portion of life which is not revealed to consciousness, he knows nothing. Consciousness does not reveal even its own substratum. Indeed, how could it do so? This would require a consciousness behind consciousness *ad infinitum*. Mind, whatever it may be, only manifests itself to us in consciousness. As the eye sees, but does not see itself, so the mind knows, but does not know itself, except as knowing, feeling, and desiring. Here we would interrupt our remarks in order to point out the exact signification we assign to the word consciousness.

Consciousness means the same thing as knowing. To be conscious .and to know are equivalent expressions. Consciousness is the *summum genus* which embraces all the cognitive, thinking, or knowing operations of the mind. Sir William Hamilton says that, "Consciousness comprehends every cognitive act; in other words, whatever we are not conscious of, that we do not know." Some persons give to the word a much fuller, and, we must say, a most objectionable signification. They speak of consciousness as feelings and sensations, of consciousness as knowledge, and of consciousness as will. Consciousness is simply knowing. Feeling, sensation, will, only exist for us in so far as they are known. Their common element, that which correlates them all, is consciousness. That in which they differ from each other is not consciousness, but its object. When I will, *I know* that I will—that is, I know an object. When I have an emotion, *I know* that I have an emotion—that is, I know an object.

To resume: As the eye sees, but does not see itself, so the mind knows, but does not know itself, save as knowing, feeling, and desiring. We may now add, that it only feels and desires, when it knows that it does so. Knowledge—consciousness—is the common element in all manifestations of mind. Now, Psychology, we have seen, treats exclusively of mind; and mind, in common with everything else, only exists *for us* through the medium of consciousnesss. Between us and being of every possible kind, immovably stands, knowing. Without the intervention of consciousness, there is nothing *for us* but a blank nihilism. We see all things through this window, and if this window be blocked up, we are personally annihilated. Nothing, then, can know mind but mind conscious. But again, nothing within the infinite universe can know anything whatever but mind conscious. Now, here we have two kinds of knowing; first, the mind knowing itself as conscious, feeling, and desiring; and secondly, the mind knowing something else than itself, namely, non-mental objects internal or external. Psychology confines itself—1, to mind as conscious, which is known by reflection; 2, to mind as feeling and desiring, which requires inward transitive knowing; 3, to organic affections, which also require inward transitive knowing. When we say that the mind knows itself, the verb to know is, as it were, a reflective verb; but when we say that the mind knows an object—whether it be mental, organic, or extra-organic—the verb to know is used as a transitive verb. Hence we say that the mind knows itself, as a thinking agent, by reflection; as feeling and desiring, by inward transitive knowing.

Here we are led to consider the objection, that those who prosecute a reflective, and inward transitive research, confine themselves to the facts of consciousness, and disclose little or nothing about life, or about the automatic functions of the body. They are constrained to confine themselves within these limits, and therefore it is, that the psychological method is so especially a divisive one. The fact is, they who make this objection are reprimanding psychology for not doing that which pertains exclusively to the province of physiology. We can imagine Physiology saying to Psychology, 'Why, you have been all this time following a wrong method; no wonder that you have met with such bad success. You have committed endless blunders, sufficient to convince any one who is open to conviction, that he had taken the wrong road. As for me, I have made some mistakes, it is true, but then I have not had the opportunities that you have. Renounce the reflective method; it never has led, nor ever will lead to fruitful results. How can a man, by merely pondering over his own thoughts, know the mind of *all* men? Such a thing is clearly impossible.'

Psychology replies—'I have not the least desire to continue old feuds, but I cannot consent to relinquish one tittle of my rightful dominions to you. My dominion is the mind as conscious. Of the connexion between life and thought, I know very little. Of "latent consciousness," of course, I know nothing whatever. Neither do I know anything about the automatic processes of life, or about the connexion between mind and brain. These things are entirely out of my sphere. All that is ascertainable by reflective, and by *inward* transitive, knowing pertains to me. All that is to be known about life and organization by means of *outward* transitive knowing pertains to you. Neither of us can act for the other. We can keep from henceforth on very good terms, and indeed it is highly essential to the advancement of the good cause to which we are devoted that we should do so. But as to my relinquishing the introspective method, that is, in fact, giving place entirely to you, that were indeed to retrograde towards the very dawn of speculation. No, I have a work to complete; so have you, and a difficult work you will find it, quite sufficient to tax your utmost energies, I can assure you. I can know nothing of your province. You can know nothing of mine. What you do pretend to know about it, you filch from me.'

Now, so that we may understand more clearly what is the province of these rival methods, we must endeavour to point out that there are two great orders, the order of knowledge, and the order of existence. The first starts from consciousness, and from this centre radiates towards the objects of consciousness. The second starts from the atomic theory, say, or from the simple proceeds to the complex. In modern times, the order of knowledge calls to mind such names as Descartes, Spinoza, Leibnitz, Berkeley, Reid,* Kant, Fichte, Schelling, Hegel, and Hamilton. The order of existence calls to mind such names as Bacon, Hobbes, Locke, Hume, Condillac, Hartley, Darwin, Brown, Comte, and J. S. Mill, in connexion with the laws of thought, and in connexion with physical science, the names of all the great natural philosophers. To this order physiologists also adhere. In the order of knowledge, the idealist seeks, principally, for the criteria of truth. He desires to know that which cannot be doubted. He seeks for indisputable principles, by means of which to arrive, deductively, at all truth whatever. He, as we have already stated, discovers certain principles which he thinks are indubitable. From these he proceeds, deductively, to derive an imposing system, which turns out to be highly improbable and forbidding. In the order of existence, on the other hand, the philosopher has not the same intense desire to discover the basis of certitude, but relying upon the spontaneous convictions of the mind, he seeks to construct a system of objective truth by observation and induction; in short, by the *à posteriori* method, in physical science, the method to which the mind cannot fail to be led. The reason to be assigned for the great success which has attended the efforts of the *à posteriori* philosophers in the discovery of physical truth, is the fact, that they have searched for accessible truth, and have found it. The rival school are more ambitious. They desire to know what truth in the abstract is—the most difficult of problems, and as yet unsolved. They disdain

---

* Philosophers of this school are divided into those who adopt the rationalistic method, a method which culminates in Hegel; and those who adopt the psychological or inductive method. Among these we may mention Reid, Stewart, Royer-Collard, and Cousin.

sense and experience, which only reveal phenomena. They would know what things *are*, not what they appear to be. They assert the superiority of reason over sense and experience. Some persons, arguing fallaciously from the past to the future, conclude that this school are wholly in error, that they aim at the unattainable. Disdaining to procure the gold that is to be found, the *à priori* philosophers are for ever making enormous, but unavailing, efforts to discover the philosopher's stone. In this ambitious attempt they must inevitably fail. Still they are as much needed as the opposite school. Idealism is the centrifugal force which keeps sensational tendencies from gravitating too abruptly towards purely animal and material principles.

The order of knowledge was, by Descartes, concisely expressed in that famous proposition, *Cogito, ergo sum.* The counter order is expressed by the converse of this proposition, namely, *Sum, ergo cogito.* Consciousness is the cause of my knowing that I exist, and indeed that anything else does. Because I am conscious, therefore it is that I *know* that I am, that I exist to myself. But the cause of my consciousness is my existence. Because I *am*, therefore it is that I am conscious. These two orders completely harmonize with each other; and never was a greater mistake made, than to imagine that one or the other must be a fiction. Although consciousness is said to be both prior and posterior to existence, it is in quite different senses that this declaration holds true. In the order of knowledge it is prior—in the order of existence it is posterior to being. Again, being, in the order of existence, is prior to consciousness—but, in the order of knowledge, posterior to it. Because I am conscious, therefore it is that I *know* that I am.

Idealism results from taking a one-sided view of human thought, from regarding perception, for example, in the order of knowledge exclusively, when it claims to be considered equally as much in the order of existence. When perception is thus viewed in its integrity, idealism is found to be a true doctrine only when it has its equipoise in realism. A complete philosophy must find room for both.

But the idealist will only accept his own half of the whole system. How is he to be convinced of his error? He must be reminded that consciousness is veracious, and that it declares that in outward perception there is an object; that this object, in the order of existence, is prior to the cognition of the same; and that, since it is prior to the cognition, it must have an existence which is entirely independent of man's existence. The Earth! Does man depend upon it, or does it depend upon man? The consciousness of every man, woman, and child replies that, of course, man depends upon the earth; and that reply of consciousness is veracious. Idealism, therefore, as an exclusive system, is untenable. It is a half-truth endeavouring to usurp the place of a whole truth, which is, that, in the order of knowledge, the cognitive faculties of man are the condition of the world's existence for him as a conscious being; but that, in the order of existence, the world is both a condition of his existence in it, and of his knowledge of it.

But what affords strong evidence of the one-sidedness of idealism, is the opposite doctrine of materialism which it has to encounter. If idealism, when elevated into an exclusive system, is abused, what shall we say of the attempt to ignore the science of mind altogether? Yet this is what Comte has endeavoured to do. He has contemptuously overlooked that which enabled him to know that there was such a being as himself, and a world in which he lived and moved. How do we realize the existence of objects, say, of material objects, since it is with these that Comte has chiefly held communion? Through the intervention of consciousness. Between us, as conscious agents, and being, irremovably stands knowing. Whatever being is *quoad nos*, it is through consciousness that it is that. Of the two, then, consciousness is more evident than its objects. This is emphatically indicated by the fact of so many gifted men, when they devoted themselves to a reflective scrutiny of their own minds, coming to the conclusion that the *non-ego* is only a modification of the *ego*. Verily, the man who *thinks* that there is no science of *thought*, is attempting to scuttle the very boat which carries him. But what is it that gives rise to this one-sided doctrine, this anti-idealism? A cause kindred to that which leads to idealism, the exclusive contemplation of a half truth, the order of existence. The counter order of knowledge, because of the absence of all reflective research, is unheeded. Nature is necessarily studied by the direct or transitive operation of the intellectual powers; and man, by the positivist, is studied in the same manner. The human mind is thought capable of being known exclusively by the physiological method. A reflective research leads to metaphysics. Metaphysical doctrines characterize the second stage of knowledge. In the positive or final stage there can be no metaphysics.

Mr. J. S. Mill, notwithstanding his strong *à posteriori* bias, is more far-seeing than Comte in this subject. Indeed, how could the author of the "System of Logic" describe mental processes so ably as he has done in that famous work, and yet hold with the French philosopher that there can be no science of mind? The thing appears to be impossible. Mr. Mill says:—

"But after all has been said which can be said, it remains incontestible by M. Comte, and by all others, that there do exist uniformities of succession among states of mind, and that these can be ascertained by observation and experiment. . . . . The successions which obtain among mental phenomena do not admit of being deduced from the physiological laws of our nervous organization; and all real knowledge of them must continue, for a long time at least if not for ever, to be sought in the direct study, by observation and experiment, of the mental successions themselves. Since, therefore, the order of our mental phenomena must be studied in those phenomena, and not inferred from the laws of any phenomena more general, there is a distinct and separate science of mind."

Now, what if we, for the sake of argument, and in further illustration of the psychological method, were to adopt Fichte's position, and say, we shall admit nothing as absolute knowledge which has the least tincture of faith in it. True science is that which, starting from absolute certitude, proceeds from certitude to certitude until the end is reached. It is plain that, if we adopt this position, we must, in the first place, settle what is the basis of truth. This, we find, Descartes has done for us. Consciousness is the only basis of certitude. Hence we perceive that, in a strictly scientific research—namely, that which seeks in the first place to discover what science in the abstract, what the science of sciences is—we must take our departure from consciousness as the only truth-standard, and adopt Descartes' proposition, *Cogito, ergo sum*, a proposition which some might pronounce to be contrary to experience. What kind of experience? That which is according to the order of existence. But then, there is another kind of experience, that which is according to the order of knowledge, and contrary to that experience, Descartes' proposition most decidedly is not. In the order of knowledge, or *quoad nos*, consciousness stands between us and all existence. Consciousness, therefore, is a most obtrusive principle, an analysis of it is all-important; and it concerns us most seriously to ascertain its laws as *the* standard of truth,* so that whether its deliverances constitute experience according to the order of existence, or according to the order of knowledge, we may be able completely to test their validity.

Since one of the charges brought against psychology is, that it neglects to investigate the laws of life, it is highly important to ascertain how far consciousness is immediately cognizant of life, for that portion of life of which we are unconscious is out of the province of psychology.† Known life commences with the fundamental and kindred perceptions. There is an act of perception which holds a highly important office among intellectual powers. Its importance arises not from its dignity, but from its fundamental and abiding character, as forming the basis of every other cognitive act. A cessation of this perception involves a cessation of every

* These laws are sketched in *The A B C of Thought: Consciousness the Standard of Truth.* Williams and Norgate.

† A man has no immediate or presentative knowledge of any life but his own.

conscious act of mind. This elementary perception seems to be that of ourselves as possessed of extended animation. As a perception it consists of two elements—1, the cognition; 2, of an object, namely, extended life. Other cognitive operations come and go, they are not of long continuance, but as long as we experience any of them, we must be experiencing this. And while internal observation leads us to this result, reason also concludes that since all sensations, with the exception of that which is the object of this perception; since all our own mental operations intermit with more or less frequency, they must necessarily involve a sensation which is abiding, within the extension of which they must have their habitation. Without this fundamental perception, all other acts of consciousness, on the supposition of their existence being possible, would be isolated from each other, like so many unlinked railway carriages. The fundamental perception, therefore, forms the basis of our personality, and the bond which unites our other powers into one whole, forming, not the man as unrealized in consciousness, and which in the order of existence precedes thought, but the man as realized in consciousness, as a personal being. Minus consciousness nothing exists *quoad nos*, we do not even exist to ourselves. Our personality, such is the evidence we possess, is a whole made up of many parts. But, like a piece of music written in a single key, it is a whole which combines unity with diversity. Of this whole, the element which forms the bond of union is the ever-fixed perception which we are contemplating. This performs the same office among cognitive acts as the involuntary system does among vital acts. Upon this fundamental perception, or presupposing it, we have other more or less variable manifestations of consciousness, those which are nearest the base being more permanent than those which are considerably removed from it. For it is a law of nature, that the most simple, the most general, is the most fundamental and abiding. The superincumbent, in proportion to its remoteness from the base, is ever tending to greater variableness and complexity, and to less generality and permanence. The crust of the earth remains, although not only countless generations of men, but even mighty cities have vanished in succession from it.

There is a question of very great importance touching the intellectual operations, namely, unconscious cerebration, to which certain mental physiologists lay claim as coming exclusively within their province; and if there be such a process, to their province it undoubtedly belongs, for what is out of consciousness is out of psychology. But is there such a process? One great objection to this theory is, that if the process in question be out of consciousness—that is, out of knowledge—its existence can never be more than a hypothesis. If the brain further the progress of knowledge without our being made privy to the act, how can we know that it performs this office ? The upholders of the theory reply, "By finding that you possess more clear, better digested, and more advanced notions of a subject, after discontinuing the study of it for a time, than you did at the moment when you ceased to apply your mind to it. You have given your mind rest, but while your mind has been resting, your brain has been busily engaged. You are perfectly aware that, for a time, you have avoided thinking of the subject, but now when you address yourself afresh to it, you find that you possess more clear notions of it, than you did at the period when you withdrew your attention from it. How is this result to be accounted for, but by the theory of unconscious cerebration ?"

Now, to all theories grounded exclusively on negative evidence, there is always this insurmountable objection, namely, that while there yet remains the slightest possibility of a positive explanation of the fact which is sought to be explained, such theories can never be fully established. If James is either in this house or the next, the mere failure to find James in the first house, before it has been completely searched, is no proof that he must be in the second house. Until, then, all positive explanations of the fact of the unconscious growth of knowledge in the mind have been actually exhausted and proved inconclusive, the mere negative evidence cited in favour of the theory under review, can never establish that theory on a permanent basis.

Well, is it possible to afford a positive explanation of the fact under discussion ? Remember, that to attribute it to an unconscious process, to unconscious cerebration, is to admit that, in a positive sense, the fact cannot be accounted for. If it can be positively accounted for, then, of course, the theory of unconscious cerebration is a pure fiction. We are merely desirous of stating the question clearly, we do not profess to be able to afford the true interpretation; but, perhaps, we may be allowed to suggest something on the point.

Our own experience is, that after intense application to a subject, the mind becomes weary and confused. The subject has then, from sheer inability to proceed successfully with it, to be laid aside. Now, it seems that, in the course of study, impressions had been made on the retaining portion of the mind; but the thinking or working portion being wearied, could not, without difficulty, attend to these impressions. To the jaded musician the finest music ceases to be a witchery of sweet sounds. When, however, after the mind had recovered its fatigue, the subject was resumed, the power to attend to the impressions which have been alluded to was at its height.

It is also to be borne in mind, that any subject which we happen to study is composed of parts among which difference and resemblance obtain in an inverse ratio. The greater the difference, the less is the resemblance; and the greater the resemblance, the less is the difference. In proportion as resemblance predominates over difference, in that proportion the resembling parts are being impressed on the memory. But in proportion as difference exceeds resemblance, in that proportion, because of their less frequent repetition, the differing parts are being less deeply impressed upon the memory than the resembling parts.* Thus, in learning to read, the parts most frequently met with are the letters of the alphabet, because it is among these that resemblance preponderates. The parts less frequently recurring, because among these difference more largely obtains, are the rarer words. From this law it follows, that a subject, as it gradually decays from the memory of the jaded student, grows more lucid, confusion gives place to order. The differences, owing to their less frequent occurrence, and the consequent light impression which they make on the memory, rapidly disappear. By this disappearance they leave the resemblances, which form the outline of the subject, more bare to view. The consequence is, that the resemblances become more intelligible. The differences, especially to the weary and confused mind, and before the outline of the subject has been fully possessed, have an obscuring effect. When, however, the differences wax fainter, and the mind recovers its full vigour, it beholds the subject much more clearly. Then the eye was dim, and the landscape obscured by mist: now the eye is keen, and so clear is the air, the hills seem close at hand.

---

### Art. IV.—SLOW AND SECRET POISONING.

* The only exception to this is, when from the extreme uncommonness of something, it makes at once a deep impression on the memory. Usually, however, the depth of an impression is in proportion to the frequency with which the impressing cause operates on the mind.

